# Radiation Response in the Tumour Microenvironment: Predictive Biomarkers and Future Perspectives

**DOI:** 10.3390/jpm11010053

**Published:** 2021-01-16

**Authors:** Niall M. Byrne, Prajakta Tambe, Jonathan A. Coulter

**Affiliations:** School of Pharmacy, Queens University Belfast, Lisburn Road, Belfast BT9 7BL, UK; n.byrne@qub.ac.uk (N.M.B.); tprajakta05@gmail.com (P.T.)

**Keywords:** biomarkers, immune infiltrate, radiotherapy, stroma, tumour microenvironment

## Abstract

Radiotherapy (RT) is a primary treatment modality for a number of cancers, offering potentially curative outcomes. Despite its success, tumour *cells* can become resistant to RT, leading to disease recurrence. Components of the tumour microenvironment (TME) likely play an integral role in managing RT success or failure including infiltrating immune *cells*, the tumour vasculature and stroma. Furthermore, genomic profiling of the TME could identify predictive biomarkers or gene signatures indicative of RT response. In this review, we will discuss proposed mechanisms of radioresistance within the TME, biomarkers that may predict RT outcomes, and future perspectives on radiation treatment in the era of personalised medicine.

## 1. Introduction

Radiotherapy (RT) is a primary treatment modality for a number of cancers, offering potentially curative outcomes [1]. Radiation treatment modalities have significantly improved over the last two decades with the introduction of advanced techniques including stereotactic radiotherapy (SRT) and enhanced imaging methodologies to improve the precision of RT delivery, thus limiting damage to healthy tissue. However, despite these advancements, resistance to radiotherapy still occurs, resulting in disease recurrence. Characterisation of radioresistance has traditionally focused on the effects of RT on tumour *cells*, overlooking the impact on supporting stromal and immune *cells* that make up the tumour microenvironment (TME) [2]. Although components of the TME have been shown to regulate angiogenesis [3] and promote malignant progression and metastasis [4], their role in the response to RT and their contribution to radioresistance is less well characterised [5]. As such, a greater understanding of the TME response could identify predictive biomarkers indicative of RT success or failure.

Predictive biomarkers offer an approach for stratifying patients who will respond favourably to a particular treatment, in turn sparing those for whom the modality may be less effective. While radiotherapy is intrinsically a precision treatment, directed to the specific architecture of the patient’s tumour, it has so far lacked a personalised approach, taking into consideration patient-specific genomic alterations or TME composition, factors that could predict the outcome of radiotherapy [6,7]. In this review, we summarise some of the recent advances in understanding the TME response to ionising radiation. In particular, we discuss the effect of radiotherapy on the tumour stroma and immune response, and how this may contribute to radioresistance. This review will also consider the biomarkers or gene expression signatures that have been developed to predict radiation outcomes. Lastly, we conclude by exploring how these approaches could be used to develop personalised radiotherapy treatment plans to improve patient outcomes.

## 2. Radiation Response in the Tumour Microenvironment

**Figure 1:**
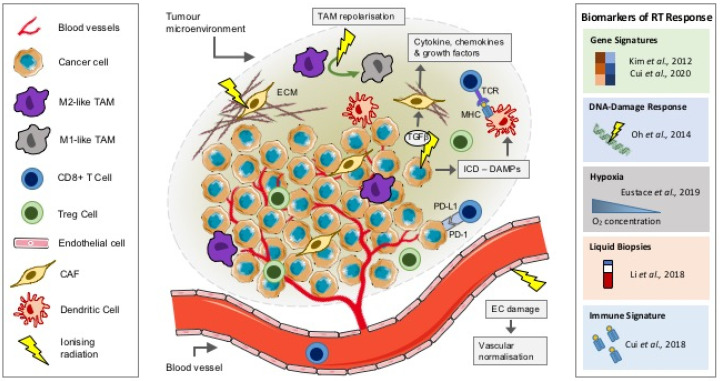
The effect of radiation on the TME. Schematic showing the role of ionizing radiation on components of the TME and predictive biomarkers of radiation response. DAMPs, damage-associated molecular patterns; EC, endothelial cell; ECM, extracellular matrix; ICD, immunogenic cell death; MHC, major histocompatibility complex; PD-1, programmed cell death protein 1; PD-L1, programmed death ligand-1; RT, radiotherapy; TAM, tumour-associated macrophage; TCR, T-cell receptor; TGFβ, transforming growth factor beta; TME, tumour microenvironment.

RT can be a cure for many; however, for some patients, the treatment fails or resistance occurs. Though ionizing radiation can induce DNA damage in tumour *cells*, a potential barrier to the success of RT may be its effects on the other components of the local TME, including the vasculature, stroma and the immune infiltrate (Figure 1). These components can influence tumour progression and response to treatment. Understanding how they are influenced by RT may be critical in predicting disease outcomes. Extracellular vesicles (EVs) including exosomes have also been shown to play a role in cancer progression, immunomodulation and importantly, in modifying the response to radiation; key examples of which are below. However, recent detailed articles focusing on the role of EV-modulated radiation response exist; as such, EVs will not form a primary focus of this review [8,9].

### 2.1. Tumour Immune Microenvironment

Immune evasion, the process by which tumour *cells* can avoid immune recognition and destruction, has become one of the hallmarks of cancer [10]. Subsequently, more recent therapeutic developments have focused on shifting the TME from an immunosuppressive environment to an immune-activated one through the use of immunotherapeutics: treatments that can effectively remove the brakes on immune signals mounting an anti-tumour response. RT has been shown to have contradictory immunomodulatory effects, influencing both proinflammatory and immunosuppressive responses, which likely influence response to treatment [5]. The inflammatory milieu of the TME, or the tumour immune microenvironment (TIME), is composed of T *cells*, natural killer (NK) *cells*, dendritic *cells* (DCs) and tumour-infiltrating myeloid *cells* (TIMs) including tumour-associated macrophages (TAMs), myeloid-derived suppressor *cells* (MDSCs) and dendritic *cells* (DCs), all of which are recruited into the TME through altered chemokine and cytokine signalling [11]. The extent and relative proportion of immune infiltration can also influence the response to treatment and progression. Tumours can be broadly separated into two categories based on their TIME: those that are immune “hot”, being infiltrated with T lymphocytes; and those that are immune “cold”, with poor infiltration [12]. In immune “hot” tumours, regulatory T *cells* (Tregs) and TAMs cooperate to support the immunosuppressive TME and may be more susceptible to the immunomodulatory effects of radiotherapy [13]. Furthermore, these immune-inflamed tumours, including non-small cell lung cancer and melanoma, are more likely to respond favourably to immune checkpoint inhibitors in comparison to immune “cold” tumours, including pancreatic and prostate tumours [14]. Lack of tumour antigens, defects in antigen presentation and poor T-cell homing to the TME by the stroma may all contribute to a “cold” tumour immune phenotype; mechanisms to modulate immune infiltration and turn these tumours “hot” could improve response to therapy [14,15,16].

The ability of radiotherapy to modulate systemic immune responses may contribute towards the observations of tumour regression at non-irradiated sites, an effect described as an abscopal response. Abscopal effects are particularly relevant when RT is combined with immune checkpoint blockade. In preclinical syngeneic models of prostate cancer, a combination of radiotherapy (20 Gy in two fractions) with antibodies against programmed death-1 (anti-PD-1) or programmed death ligand-1 (anti-PD-L1) (iRT) significantly increased median survival (70–130%) in comparison to anti-PD-1 monotherapy, contributing to an abscopal response in which the unirradiated tumours responded similarly to the irradiated tumours. Importantly, this effect was shown to be mediated through antitumour CD8+ (cytotoxic) T *cells* [17]. Clinical observations of the abscopal effect have been rare in radiation oncology; however, with the development and advancement of immunotherapeutics, these observations are becoming more frequent across a variety of tumour types [18]. Clinically, in patients with unresectable melanoma combining anti-PD-1 therapy with hypofractionated RT (typically 26 Gy in 3–5 fractions) resulted in abscopal treatment responses in 36% of patients [19,20]. Targeting of another immune checkpoint, cytotoxic T-lymphocyte antigen 4 (CTLA-4), with the monoclonal antibody ipilimumab in combination with RT has also been shown to result in abscopal responses both preclinically in models of breast cancer and clinically in melanoma and lung cancer patients [21,22,23,24]. Interestingly, EVs isolated from irradiated tumour *cells* (H22 cells and 4T1 *cells*; 8 Gy) in vitro were shown to have immunomodulatory effects when mice were inoculated in vivo, enhancing CD8+ and CD4+ T-cell infiltration in lung metastasis in comparison to nonirradiated EVs [25]. Dose and fractionation are likely to play a critical role in the immunological responses to RT; however, the molecular and cellular mechanisms underpinning this immune-priming effect are still poorly understood [26].

RT-induced cell death is typically thought to occur through DNA damage, particularly in the form of double-strand breaks (DSB). Subsequently, the tumour cell response to radiation-induced DNA damage (RIDD) is dependent on its DNA damage response (DDR), which can activate downstream signalling to repair damage, thus contributing to radioresistance [27]. While the immune cell compartment, including lymphocyte and myeloid populations, may be more resistant to RIDD, RT can modulate immune signalling within the TME, promoting immune cell recruitment and activation and triggering immunogenic cell death [28]. RT-induced immunogenic cell death results in a cascade of events, starting with the release of damage-associated molecular patterns (DAMPs) (Figure 1) [29]. These “danger” signals released by tumour *cells* include high-mobility group box 1 (HMGB1) and ATP, triggering innate and adaptive immune responses through the expression of major histocompatibility complex (MHC) class I and MHC-II molecules. These antigen-presenting *cells* (APCs) can in turn can prime CD8+ T *cells* to induce an antitumour response [28]. In fact, RT has been shown to upregulate MHC-I expression preclinically in tumour cell lines in vivo, an observation that has been recapitulated in ex vivo-irradiated tumour biopsies [30]. Cytosolic double-stranded DNA (dsDNA) released as a result of RIDD can also promote dendritic cell activation through guanosine monophosphate–adenosine monophosphate synthase (cGAS)/stimulator of IFN *genes* (STING)/interferon (IFN) signalling, leading to CD8+ T-cell activation [31].

TIM populations, including TAMs, form another important component of the TIME and although they have a complex plasticity, they are usually organised as classically activated (M1) or alternatively activated (M2) *cells*. Numerous stimuli including chemokines can influence TAM polarisation from a proinflammatory (antitumour) M1 to an anti-inflammatory (protumour) M2 phenotype, which promotes tumour angiogenesis, tissue remodelling and tumour progression [32]. Interestingly, the frequency of TAMs has also been associated with clinical treatment response and disease progression [33,34]. In murine tumour models, low-dose gamma irradiation (LDI; 2 Gy) has been shown to promote repolarisation of M2-like TAMs towards M1-like inducible nitric oxide synthase (iNOS)-expressing TAMs, contributing to T-cell recruitment and tumour regression (Figure 1) [35]. TAMs and MDSCs are dependent on colony-stimulating factor (CSF1) signalling for recruitment into the TME. In murine models of breast cancer, blocking CSF1/CSF1R signalling inhibited TAM recruitment and delayed tumour regrowth following RT (5 Gy), an effect associated with an increase in CD8+ T *cells* and a reduction in CD4+ (helper) T *cells* [36]. Similar effects were observed following CSF1R signalling blockade in combination with RT (3 Gy, five fractions) in syngeneic models of prostate cancer in vivo. Furthermore, serum levels of CSF1 were also shown to be elevated in prostate cancer patients following RT [37]. Clinically, in patients with T3 rectal cancer, a short course of radiotherapy (neoadjuvant hyperfractionated 25 Gy in 10 fractions; surgery performed on day 2–5) promoted TAM repolarisation towards an M1-like proinflammatory phenotype. Interestingly, ex vivo modelling of this response suggested that HMGB1 in EVs from irradiated tumour *cells* could be responsible for this effect on TAM polarisation [38].

### 2.2. Cancer-Associated Fibroblasts

The stromal compartment of the TME plays an integral role in the response to treatment, including RT (Figure 1). Radiotherapy-induced tissue fibrosis is a late side effect where myofibroblast transformation leads to the excess production of collagen and deposition of components of the extracellular matrix (ECM) [39]. RT can also lead to the release of the pleotropic cytokine transforming growth factor beta (TGFβ), which can modulate fibroblast phenotype and function [40]. Fibroblasts recruited into the TME are transformed into cancer-associated fibroblasts (CAFs), where they play a role in regulating the extracellular matrix [41]. Furthermore, CAFs are responsible for the secretion of a number of cytokines (including interleukin 6 (IL6) and IL8), chemokines (including C-X-C motif ligand 12 (CXCL12)) and growth factors (including TGF-β and platelet-derived growth factor (PDGF)) that can influence immune cell fate and tumour progression, often contributing to the immunosuppressive TIME [42]. However, the effects of RT on the stromal compartment of the TME including CAFs are less well understood and they appear to have contradictory roles, contributing to both tumour growth and suppression [43]. Coimplantation of A549 lung tumour xenografts with preirradiated CAFs (at both 18 Gy × 1 fraction or 6 Gy × 3 fractions) abrogated the protumour growth effect observed in tumours coimplanted with nonirradiated CAFs [44]. In contrast, irradiated fibroblasts (1, 6 or 12 Gy) have been shown to express high levels of TGF-β1 and promote human T3M-1 squamous cell carcinoma (SCC) invasion and growth [45]. Furthermore, EVs derived from CAFs were shown to contribute to colorectal cancer cell stemness and radioresistance (6 Gy) in vitro, through the activation of the TGF-β signalling pathway [46]. It is therefore clear that more work is needed to understand the complex role of CAFs in the tumour response to RT.

### 2.3. Tumour Vasculature

The integrity of the tumour vasculature differs significantly from that of physiologically normal vessels, characterised by abnormal recruitment of pericytes, leading to increased tortuosity and porosity. This, in part, contributes to treatment failure through poor drug penetration into the TME, establishing local hypoxia gradients and increasing the yield of reactive oxygen species [47]. The effect of RT on the tumour vasculature has been well studied, with tumour blood vessels and their endothelial *cells* proven to exhibit increased sensitivity to radiation, a response likely dependent on total radiation dose and fractionation schedule [5,48,49]. Vascular damage is mainly witnessed at radiation doses exceeding 5 Gy. Conversely, individual, low-dose fractions have been shown to temporarily stimulate blood flow, while at higher or cumulative doses, the vascular network is disrupted, promoting hypoxic stress that can trigger tumour cell death [50,51]. In a recent dose-escalation study, single administration of 2, 4 or 8 Gy doses were shown to compromise the tumour vasculature in a dose-dependent manner, prolonging the survival of mice bearing CT-2A (high-grade glioma) tumours. Interestingly, this was also associated with changes in the TIME, promoting an increase in CD8+ T *cells* and a reduction in M2-like TAMs [52]. Potiron et al. [53] reported that RT (at both 10 × 2 Gy and 2 × 12 Gy) induces tumour vasculature normalisation and remodelling, thus improving the distribution and efficacy of the anticancer drug doxorubicin (DOX) [53]. Further evidence of the effects of RT effects on endothelial cell permeability has been demonstrated in vitro. Monotherapy radiation doses to primary human umbilical vein endothelial *cells* (HUVECs) increased permeability and transmigration of tumour *cells*, owing to altered metalloprotease ADAM10 expression and degradation of VE-cadherin, both of which play an integral role in maintaining intercellular junctions and vascular integrity [54]. High radiation doses (>20 Gy) were also found to cause transient endothelial dysfunction, platelet leukocyte adhesion and increased expression of hypoxia-inducible factor-1α (HIF-1α) in pancreatic tumours [55]. However, a recent study indicated that high-dose RT (>8 Gy) induced expression of Notch1 signalling in HUVEC monolayers. Consequently, in vivo high-dose RT, in combination with inhibition of Notch1 signalling, resulted in a significant reduction in tumour vessel endothelial cell coverage in comparison to high-dose RT alone, suggesting Notch1 signalling may protect tumour vessels from radiation-induced damage [56]. Furthermore, it is also well understood that oxygenated tumour *cells* are preferentially killed by RT, due to oxygen-induced fixation of radiation-induced DNA damage. However, this effect has been proven to accelerate the production of proangiogenic cytokines, inhibiting treatment-induced apoptosis, stimulating a postradiotherapy angiogenic burst that can contribute to eventual tumour regrowth [57].

## 3. Predictive Biomarkers of Radiation Response

Precision medicine based on common tumour-specific alterations, emerging from high-throughput molecular profiling, has become a reality in recent years. This approach underpins the discovery of clinically validated prognostic and/or predictive biomarkers, allowing for stratification of patients based either on those most likely to derive benefit or have treatment-related harm limited. This strategy gained significant momentum in the chemotherapy field with the development of various commercially produced kits such as Prosigna (NanoString Technologies, Inc., Seattle, USA) and MammaPrint (Agendia, Amsterdam, The Netherlands), designed to aid clinical decision-making [58,59]. However, equivalence in radiotherapy has not yet been achieved due to the variability in radiation response, an effect attributed to tumour heterogeneity. Heterogeneity is an umbrella term used to describe both intra- and intertumour variability at the morphological, physiological and more recently, genetic levels. Divergence of these features exerts a profound influence on localised factors such as vascular integrity, tumour oxygenation and immune infiltrate, ultimately influencing treatment outcome (detailed in Section 2 [5,13,48]). In an effort to address the issue of heterogeneity, research efforts have shifted from focusing on macroscopic phenotypic or environmental variation to the identification of commonality at the molecular level. Table 1 provides an outline of biomarkers for radiotherapy response in a number of tumour types (summarised in Figure 1); these are discussed further in the sections below. 

### 3.1. Gene Signatures of Radiation Sensitivity

An early example of this approach used the clonogenic assay to profile radiation sensitivity, based on survival fraction data at 2 Gy (SF2), of the NCI-60 cancer cell line panel [60]. This was then correlated against gene expression data from four published microarray platforms, identifying significant alterations in expression profiles for 31 *genes*, common to each microarray dataset. Unsurprisingly, significant suppression of *genes* which regulate cell cycle progression (*CCNA2*, *CDK6*, *CCND1)* and DNA damage repair were associated with increased radiosensitivity. *CCND1*, the gene encoding for cyclin D_1_, stalls cell cycle progression, providing time for DNA damage repair, ultimately suppressing radiation-induced apoptosis [72]. Therefore, suppressed *CCND1* and other cell cycle regulatory *genes* may contribute, in part, towards a genetic signature for identifying radiosensitive tumours. A second set of *genes* common to the top 10% most radiosensitive (SF2 < 0.2) *cells*, and totally absent from the most radioresistant (SF2 > 0.8), were those involved in integrin signalling, cell adhesion and cytoskeletal remodelling. Cell-adhesion complexes and integrin signalling act both directly and indirectly to influence radiation response [73]. Cell-to-cell contact and adhesion with the extracellular matrix are central features of the protumour phenotypes of migration and invasion. Along with integrin β1, the 31-gene profile identified downregulation of *ITGB5*, the gene encoding integrin β5, as a highly significant indicator of radiosensitivity [60]. Indeed, radiosensitisation achieved through the antagonism of αvβ5 integrin using a cyclic-RGD (arginine-glycine-aspartate) containing peptide was the focus of a large phase III clinical trial for the treatment of glioblastoma multiforme [74]. This was based on the rationale that αvβ5 antagonism suppresses tumour angiogenesis and metastasis, an effect in part attributed to the dampening of major cancer-related signalling pathways, including *Wnt* and *PI3K* [75]. Developed as a universal predicator of radiation sensitivity, independent of tumour type, many of the 31 *genes* identified likely hold predictive value in relation to radiation response. However, stringent application using only the most radiosensitive or radioresistant *cells* again highlights the problem of heterogeneity, where 80% tumour models analysed exhibited intermediary gene expression alterations, diluting the predictive power of the signature.

Recent approaches adopting a similar strategy tend to focus on a specific disease type. Breast cancer radiotherapy is most commonly used in the adjuvant setting to improve treatment outcomes, forming a core strategy of breast conservation surgery and mastectomy. However, not all patients benefit from adjuvant radiotherapy and some experience significant debilitating late effects [76]. The importance of identifying those who will benefit most from adjuvant radiotherapy was neatly demonstrated in a study using FFPE tumour tissue from the Danish Breast Cancer Cooperative Group (DBCG82bc) cohort. Applying a seven-gene signature to stratify patients into either high-risk loco regional recurrence (LRR) or low-risk LRR, the authors were able to establish that postmastectomy radiotherapy would benefit only those identified as high risk, providing no benefit to low-risk patients [61]. Adopting a similar strategy to the 31-gene signature, Speers et al. [62] correlated the radiation sensitivity (SF2) of a panel of breast cancer models against gene expression changes, developing a radiation sensitivity signature (RSS), which was subsequently shown to be the most significant factor in prediction of loco-regional recurrence, beating all clinicopathologic features used in clinical practice [62]. While a clear step forward, RRS remains a prognostic signature for loco-regional control, and not predictive of radiation response. Similar predictive gene signatures have been developed, including a six-gene signature (including *genes* such as HOXB13 and NKX2-2) that was also shown to predict radiotherapy sensitivity in breast cancer [77]. Applying a 24-gene signature to prostate cancer patients who had undergone radical prostatectomy to identify those most likely to benefit from postoperative radiotherapy similarly found that those with a high PROTOS (postoperative radiation therapy outcomes score), indicative of radiation-sensitive tumours, significantly benefited from radiotherapy, with a 10-year metastasis rate of 4% (95% CI 0–10) versus 35% (CI 7–54) for those not receiving radiotherapy. However, in the low PROTOS score group, radiotherapy proved detrimental (HR 2.5 (CI 1.6–4.1); *p* < 0.0001) in the 157-patient cohort training group and of no benefit in the 248-patient validation cohort [63]. Liu et al. [64] recently used multiple omics data to develop a prediction model of sensitivity to radiation in head and neck squamous cell carcinoma (HNSCC) tumours. A 12-gene signature was established from differentially expressed *genes* in patients treated with or without RT and used to develop a scoring system. Those HNSCC patients with a low score had a higher radiosensitivity and were shown to benefit from RT [64].

### 3.2. DNA Damage Response Biomarkers

The antitumour effects of radiotherapy are directly proportional to the degree to which potentially lethal DNA DSBs are both induced by radiation and are sustained by the cell following activation of DDR processes. Continual refinements to the delivery of radiotherapy have ensured that the DNA-damaging properties of the most commonly utilised radiation sources, such as X-rays and γ-rays, minimise dose to surrounding healthy tissue, while focusing dose on the target volume. In parallel, intensive research efforts have led to the development of numerous small-molecule inhibitors targeting key DNA damage repair proteins, thus sustaining radiation-induced damage, resulting in increased tumour cell death. This is the fundamental basis of many radiosensitising strategies. Key targets of the DNA damage response pathways for which clinically utilised inhibitors have been developed include the ATM/ATR (ataxia–telangiectasia mutated and Rad3-related) signalling pathways, PARP (poly (ADP-ribose) polymerase), DNA-PKcs (DNA-dependent protein kinase, catalytic subunit), BRAC1 (breast cancer1 C terminal) and HIF-1, amongst others. While reviewing the full therapeutic potential of these inhibitors is beyond the scope of the current article, several recent publications provide comprehensive details of this field [27,78,79]. Herein, we aim to focus on the utility of gene expression alterations in DDR *genes* as prognostic/predictive indicators of radiation response. Piening et al. [65] developed an early radiation-derived gene signature, evaluated for prognostic utility in breast cancer. The signature was derived from gene expression alterations following a 5 Gy dose across a panel of nontumour lymphoblast *cells*, a relevant point given that genomic instability in tumours support aberrant DDR activity. Expression levels of 219 *genes* were altered with 160 being induced and 59 repressed by radiation. Using a gene set enrichment algorithm [80], the prognostic utility of the signature was evaluated against publicly available breast cancer microarray data. With respect to the repressed *genes*, tumour samples neatly clustered into two groups, aligning with gene repression or not, where the former strongly correlated with increased proliferation and poor overall treatment outcomes. Similarly, *genes* induced by radiation correlated positively with those who responded favourably to radiation treatment, promoting the expression of *genes* involved in negative regulation of the cell cycle, apoptosis (e.g., caspases) and DNA damage repair proteins. Importantly, applying the same approach but using the NCI-60 cancer cell line panel to derive the radiation signature failed to discriminate between favourable and poor outcomes, with no overlap between the altered gene set signature [65]. This clearly illustrates the impact of genomic instability in influencing the DDR response and an important point for consideration in the development of radiation biomarkers.

Another study exploited the overlapping DNA damage responses activated by both chemotherapy and radiotherapy, producing a radiation-induced 30-gene signature. This signature was proven capable of discriminating between breast cancer patients likely to achieve a pathological complete response (pCR) to neoadjuvant chemotherapy and poor-responding patients. Importantly, pCR represents the most relevant clinical end point for predicting improved overall and disease-free survival [81]. In addition to *genes* clearly linked to DNA damage pathways, such as the extracellular signal-regulated kinase (*ERK*) pathway, *AKT*, *mTOR* and *NF-ĸB*, radiation significantly elevated the expression of metabolism processing *genes*, in particular *PDHA1* and *LDHB*. These genes encode for key proteins driving pyruvate metabolism and energy production, along with the catalytic conversion of pyruvate to lactate, thus indicating that tumours with a high metabolic demand are more likely to prove sensitive to the effects of chemo- and radiotherapy [66].

### 3.3. Hypoxia Biomarkers

As outlined previously, hypoxia resulting from aberrant tumour vasculature can influence RT resistance. As such, there is a strong rationale for identifying robust biomarkers of tumour hypoxia that predict response to RT [82]. Traditionally, tumour hypoxia was measured using oxygen electrode probes, endogenous HIF-1α levels, physiological markers such as pimonidazole staining or other imaging methodologies (MRI). However, gene signatures may better represent the nuances of hypoxia within the TME that might predict response to RT. To this end, Eustace et al. [67] developed a 26-hypoxia gene signature (informed by a 121-gene hypoxia meta-signature derived from datasets of head and neck, breast and lung cancers [83]) predicting treatment response in laryngeal cancer. This hypoxia signature, composed of *genes* involved in glucose metabolism (*ALDOA*, *ENO1*, *LDHA*), cell proliferation (*CDKN3*, *FOSL1*) and angiogenesis (*VEGFA*), could predict those patients receiving RT for whom hypoxia-modifying ARCON (accelerated radiotherapy with carbogen and nicotinamide) therapy would be of benefit in laryngeal carcinomas [67]. The approach of stratifying patients for hypoxic modification of RT has also been performed by Troustrup et al. [68] to classify HNSCC tumours as “more” or “less” hypoxic [84]. A 15-gene hypoxic signature including *genes* for stress response (ADM, HIG2), cell proliferation (FOSL2, IGFBP3) and glucose metabolism (ALDOA, FKBP3) was developed from HNSCC cell lines under hypoxic conditions, and subsequently validated in patients that had previously been hypoxia-evaluated [85,86]. The predictive power of this gene signature was validated in a clinical cohort of HPV-negative HNSCC tumours, with those classified as having “more” hypoxic tumours having more favourable outcomes (loco-regional tumour control and disease-specific survival) after combining RT with hypoxia modification using nimorazole [68].

### 3.4. Liquid Biopsies

Minimally invasive liquid biopsies represent an area of intense research interest. While the field is in relative infancy, with no commercially validated tests, the identification of circulating biomarkers predicative of radiation response holds tremendous potential. MicroRNAs (miRNAs) are differentially regulated in a number of disease types and following exposure to ionizing radiation; they therefore offer a potential biomarker to predict treatment response in cancer [87,88,89]. A radiotherapeutic response predication was developed for patients with lower-grade glioma (LGG), based on the expression of five miRNAs. The signature was capable of classifying those as low-risk or high-risk in terms of survival and radiation response, based on the analysis of miRNA expression profiles in 624 patients. This signature was found to be superior to isocitrate dehydrogenase (IDH) mutational status in predicting survival in LGG [90]. Of particular interest is free plasma or exosome secretion of miRNAs predictive of radiation response: Li et al. [69] linked low-level miR-221 expression with increased radiation sensitivity, a finding subsequently correlated with several patient studies reporting that low serum levels of miR-221 and miR-125b are indicative of low-risk prostate cancer [69,91,92]. Furthermore, Li et al. [70] associated the levels of three miRNAs (miR-374a-5p, miR-342-5p and miR-519d-3p) with radiation responses in the plasma of patients with nonmetastatic rectal cancer and head and neck cancers. Prediction classifiers were developed from miRNA signatures in pre- and postradiotherapy samples and could significantly distinguish between radiation responders and poor responders 6 months postradiotherapy [70]. The importance of effective biomarkers, particularly in the prostate cancer setting, is evident considering that prostate-specific antigen (PSA) screening has formed the bedrock of prostate cancer diagnosis for over 25 years—a test lacking in specificity—resulting in significant treatment related morbidities from overdiagnosis and overtreatment [93]. Given the role of the TIME in influencing tumour fate postradiotherapy (detailed in Section 2), immune infiltrate composition in the TME may predict radiotherapy response and prognosis in cancer patients [5,94]. Cui et al. [71] pioneered a combined radiation sensitivity (RS) gene signature with an antigen-presentation (AP) immune signature, establishing a dual-modality approach with predictive capabilities of radiation response. Independently, both RS and AP signatures were proven capable of predicting increased disease-specific survival (DSS) in patients identified with either radiosensitive or immune-effective tumours, with the reverse observed in radioresistant and immune-defective individuals. Importantly, integration of both signatures further strengthened the predictive capabilities of either signature used independently [71].

## 4. Conclusions and Future Perspectives

RT is the treatment of choice for a number of cancer, designed to target and kill tumour *cells*; however, it triggers a myriad of effects on other components of the TME, including the vasculature, stroma and the immune compartment [5]. The immunomodulatory effects of RT are complex, with reported changes to the proportions and functionality of T *cells* and antigen-presenting dendritic *cells*, and effects on TAM polarisation within the TME. This effect is further complicated by clinical observations of an increase in the abscopal effect reported in patients receiving RT in combination with immunotherapeutics. RT has also been shown to affect tumour vascular architecture, inducing tissue fibrosis. It is important to note that the majority of responses to RT in the TME reported above are in the context of conventional X-ray or photon radiation therapy. Recent advances in the clinical delivery of RT, including high-energy proton beam therapy and heavy ion therapy, have the improvement of delivering more dose in the Bragg peak with a lower dependence on tissue oxygenation and improved biological effectiveness [95]. While these newer treatment modalities are likely to have biological effects on the components of the TME outlined in this review, their response has been less well characterised [96,97]. Therefore, it is of critical importance to take into consideration the role of the TME when considering radiobiological responses and disease recurrence. As RT techniques have evolved over the last two decades, so too have their physical precision, aided by improved imaging guidance and technological advancements. However, genomic precision has lagged, as most RT treatment planning is designed around the tumour and local tissue architecture, with the aim to deliver the maximum dose to the tumour while sparing healthy tissue. However, as highlighted above, genomic signatures could allow for a greater prediction of those patients for whom RT would be of benefit as a single therapy or in combination with radiation sensitizers or hypoxia modifiers [6]. Yet, of critical importance, these findings further stress the necessity for a precision medicine approach, in that not only do patients with radioresistant tumours fail to experience radiotherapy benefit, but that treatment is actually detrimental both in terms of DSS and toxicities associated with radiation-induced late effects [71]. Taking a more “personalised” approach to RT could ensure patients receive the most benefit from their treatment.

## Figures and Tables

**Table 1:** Biomarkers of radiotherapy response.

	Year	Cancer Type	Biomarker	Results	Ref
**Gene signatures**	2012	NCI-60 human tumor cell lines screen	A 31-*gene* signature developed from meta-analysis of microarray data correlated with clonogenic assay data to identify radiosensitive or radioresistant *cells*	*Genes* involved in cell cycle progression (*CCNA2*, *CDK6*, *CCND1)* and DNA damage repair were associated with increased radiosensitivity	[60]
	2014	Breast cancer	A 7-gene signature applied to the Danish Breast Cancer Cooperative Group (DBCG82bc) cohort to stratify patients into either high-risk locoregional recurrence (LRR) or low-risk LRR	Identified that post-mastectomy RT would benefit only those identified as high risk, providing no benefit to low-risk patients	[61]
	2015	Breast cancer	Radiation sensitivity gene signature developed from correlating radiation sensitivity (SF2) of a panel of breast cancer models against gene expression changes	Gene signature significantly predicted loco-regional recurrence; beating all clinicopathologic features used in clinical practice	[62]
	2016	Prostate cancer	A 24-gene signature applied to prostate cancer patients who had undergone radical prostatectomy to identify those most likely to benefit from postoperative radiotherapy	Retrospective analysis identified that those patients with a high PROTOS (post-operative radiation therapy outcomes score), indicative of radiation sensitive tumours, were less likely to develop metastasis at 10 years post-RT. In the low PROTOS score group, radiotherapy proved detrimental	[63]
	2020	HNSCC	A 12-gene signature	Classified patients with a higher radiosensitivity for whom RT would be beneficial and could predict overall survival.	[64]
**DNA-damage response**	2010	Breast cancer	*Gene* expression signature associated with DDR, correlated against publicly available breast cancer microarray data	DDR-associated *genes* induced by radiation correlated positively with those who responded favourably to radiation treatment	[65]
	2014	Breast cancer	Radiation-induced 30-gene DDR signature	Gene signature was capable of discriminating between breast cancer patients likely to achieve a pathological complete response (pCR) to neoadjuvant chemotherapy and poor-responding patients	[66]
**Hypoxia**	2013	Laryngeal cancer	A 26-hypoxia gene signature	Could predict those patients receiving RT for whom hypoxia-modifying ARCON (accelerated radiotherapy with carbogen and nicotinamide) therapy would be of benefit	[67]
	2012	HNSCC	A 15-gene hypoxia signature	Classified patients who would benefit from combining RT with hypoxia modification (nimorazole)	[68]
**Liquid biopsies**	2011	Prostate cancer	Altered miRNA expression: developed through screening of miRNAs in prostate cancer *cells* (LNCaP) in response to RT	Suppressed miR-221 expression linked with increased radiation sensitivity: data subsequently correlated in clinical datasets where low serum levels of miR-221 are indicative of low-risk prostate cancer	[69]
	2018	Nonmetastatic rectal cancer and head and neck cancers	miRNA expression rations: prediction classifier	The expressions of three miRNAs—miR-374a-5p, miR-342-5p and miR-519d-3p—were significantly different between responsive and poor-responsive RT groups. miRNA classifier successfully predicted radiotherapy outcomes	[70]
**Immune signature**	2018	Breast cancer	Combined radiation sensitivity (RS) gene signature with an antigen-presentation (AP) immune signature	Both RS and AP signatures capable of predicting increased disease specific survival (DSS) in patients identified with either radio-sensitive or immune-effective tumours	[71]

## Data Availability

Data sharing not applicable.
